# Exogenous NADH enhances polycyclic aromatic hydrocarbon degradation in *Novosphingobium pentaromativorans* US6-1 by boosting energy metabolism and reducing oxidative stress

**DOI:** 10.1128/aem.00411-26

**Published:** 2026-05-20

**Authors:** Qiu Meng, Fengjiao Lv, Jiefeng Huang, Han Chen, Feifei Cao, Zhiliang Yu

**Affiliations:** 1 College of Biotechnology and Bioengineering, Zhejiang University of Technology 630365, Hangzhou, China; 2 Hangzhou Chuhuan Science and Technology Co., Ltd., Hangzhou, China; Universidad de los Andes, Bogotá, Colombia

**Keywords:** *Novosphingobium pentaromativorans *US6-1, NADH, polycyclic aromatic hydrocarbons, electron transport chain, reactive oxygen species

## Abstract

Polycyclic aromatic hydrocarbons (PAHs) are environmentally persistent pollutants that pose threats to both the environment and human health. Recently, bioremediation has emerged as a promising strategy for PAH pollution. NADH plays a crucial role in microbial metabolism and participates in PAH degradation. However, the mechanism underlying the influence of NADH on polycyclic aromatic hydrocarbon degradation remains unclear. The bacterium *Novosphingobium pentaromativorans* US6-1 degrades PAHs efficiently, and exogenous NADH supplementation promoted both cell growth and phenanthrene degradation. Promoter activity assays revealed that NADH upregulates the expression of PAH-degrading enzyme genes *salA* and *xylE* and enhanced the *in vivo* activity of the XylE enzyme. Since NADH did not act as a direct cofactor of XylE in cell-free assays, this enhancement is likely attributed to transcription upregulation and optimized intracellular conditions. Furthermore, NADH reduced intracellular reactive oxygen species (ROS) levels more effectively than antioxidant glutathione (GSH), suggesting that NADH supports cell fitness through mechanisms that extend beyond simple chemical ROS scavenging. Interestingly, NADH boosted the intracellular electron flux, and enhancing the electron transport system through either gene overexpression or FAD supplementation similarly improved PAH degradation. In conclusion, NADH promotes PAH degradation in *N. pentaromativorans* US6-1 through a multifaceted mechanism involving upregulating the key catabolic genes, enhancing *in vivo* critical enzyme activity, reducing intracellular ROS accumulation, and increasing electron flux. Our findings elucidate novel regulatory pathways of NADH in PAH degradation, thus highlighting its potential as a target for optimizing PAH bioremediation strategies.

**IMPORTANCE:**

The oxidation of polycyclic aromatic hydrocarbons (PAHs) by degrading bacteria generates toxic intermediates. Accumulation of these compounds disrupts intracellular redox balance, exacerbating cellular toxicity. Furthermore, microbial PAH degradation imposes high energy demands and depletes essential redox cofactors. Despite the critical role of NADH as a central energy currency and reductant in cellular metabolism, the specific mechanisms underlying the influence of NADH on PAH biodegradation, particularly its interplay with redox homeostasis, remain poorly understood. This study has elucidated that NADH enhances PAH degradation efficiency by alleviating oxidative stress due to PAH oxidation. This study provides insights critical for advancing engineered bioremediation solutions.

## INTRODUCTION

Polycyclic aromatic hydrocarbons (PAHs) are toxic pollutants composed of two or more aromatic rings, possessing potential hazards such as teratogenicity, carcinogenicity, and mutagenicity. Based on molecular weight differences, PAHs are categorized into two types: low-molecular-weight PAHs and high-molecular-weight PAHs. Low-molecular-weight PAHs include anthracene, phenanthrene, etc.; high-molecular-weight PAHs include pyrene, benzo[*a*]pyrene, etc. PAHs exhibit environmental persistence, resistance to degradation, and high toxicity, enabling bioaccumulation in organisms (1). PAHs not only attach to microplastic particles and disperse via water flow, posing hazards to aquatic environments, but also contaminate soil, get absorbed by plants, and consequently enter the food chain to endanger human health (2, 3). PAHs have diverse sources, primarily categorized into natural generation and anthropogenic emissions. Natural generation mainly includes volcanic eruptions and forest fires. Anthropogenic emissions primarily stem from the combustion of liquid fossil fuels, vehicle exhaust, ship fuel leaks, and aquaculture pollution (4, 5).

*Novosphingobium pentaromativorans* US6-1 is a marine bacterium isolated from muddy sediments in Osan Bay, South Korea, capable of degrading various PAHs, including naphthalene, phenanthrene, fluoranthene, and benzo[*a*]pyrene. Most genes encoding PAH-degrading enzymes in strain US6-1 are clustered on the plasmid pLA1, among which oxidases play a crucially important role in the PAH degradation process (6). Proteomic analysis has discovered that US6-1 contains various enzymes related to PAH degradation, such as 1-ketovalerate-3-monooxygenase, salicylaldehyde dehydrogenase, and polycyclic aromatic hydrocarbon ring-hydroxylating dioxygenase. The presence of these enzymes indicates that PAHs are initially catalyzed by ring-hydroxylating dioxygenase, followed by subsequent metabolic pathways. Moreover, US6-1 contains glycosphingolipids in its cell membrane and exhibits strong surface hydrophobicity, which facilitates the adsorption of PAHs and subsequently accelerates PAH degradation. US6-1 can also enhance PAH degradation capacity through strategies, such as tolerating starvation stress, downregulating the expression of transporter-encoding genes, and reducing excessive accumulation of toxic substances (7).

Currently, significant progress has been made in the research on the degradation of PAHs by US6-1. Our preliminary studies have revealed that the genome of US6-1 contains 126 PAH degradation-related genes, which encode all the enzymes required for phenanthrene degradation. During the degradation of phenanthrene, US6-1 produces an intermediate metabolite called salicylic acid, which inhibits cell growth and slows down the degradation of phenanthrene. It is noteworthy that US6-1 primarily introduces phenanthrene into the tricarboxylic acid cycle through the salicylic acid pathway. During this biotransformation process, US6-1 gradually breaks down phenanthrene into smaller molecules, which are ultimately converted into harmless substances and enter the tricarboxylic acid cycle. This transformation process involves various biocatalytic reactions, including but not limited to oxidation, reduction, hydrolysis, etc., and entails both the consumption and generation of NADH (8).

NADH is one of the most crucial coenzymes in living organisms, playing a pivotal role in various biochemical reactions. As an electron carrier, NADH serves as a vital electron donor within cells, driving redox reactions and energy metabolism by transferring electrons from reducing agents to oxidizing agents, thereby facilitating energy conversion. Furthermore, NADH also functions as a coenzyme in numerous enzyme-catalyzed reactions, enabling its conversion into NAD^+^ (9). NADH is also involved in key metabolic processes, such as glycolysis and the tricarboxylic acid cycle, and plays a crucial role in biosynthetic reactions, including the synthesis of fatty acids, amino acids, and nucleotides (10).

In the aromatic hydrocarbon degradation process, NADH primarily participates in the oxidation of aromatic hydrocarbons, converting them into corresponding phenolic compounds (11–14). As the primary carrier for electron transfer in the electron transport chain, NADH can serve as an electron donor and participate in the degradation reactions (15–18). It has been found that redox reactions of aromatic compounds generate ROS, leading to oxidative stress (19, 20). Therefore, the generation and consumption of NADH are crucial for maintaining intracellular redox balance to aromatic compound oxidation.

Phenanthrene serves as a representative contaminant for studying PAH degradation (21, 22). Through analyzing the phenanthrene degradation pathway of US6-1, it was found that NADH participates in multiple catalytic reactions of phenanthrene degradation (23), suggesting that NADH may play an important role in the phenanthrene degradation process. However, the detailed mechanisms underlying the effect of NADH on phenanthrene degradation remain unclear yet. This study for the first time revealed that NADH promotes PAH degradation in strain US6-1 through a multifaceted mechanism involving transcription regulation, mitigation of oxidative stress, and enhancement of electron transfer. Our findings highlight the crucial role of NADH in PAH degradation, providing insights critical for advancing engineered bioremediation solutions.

## RESULTS

### Exogenous NADH specifically enhances PAH utilization by US6-1

**Fig 1:**
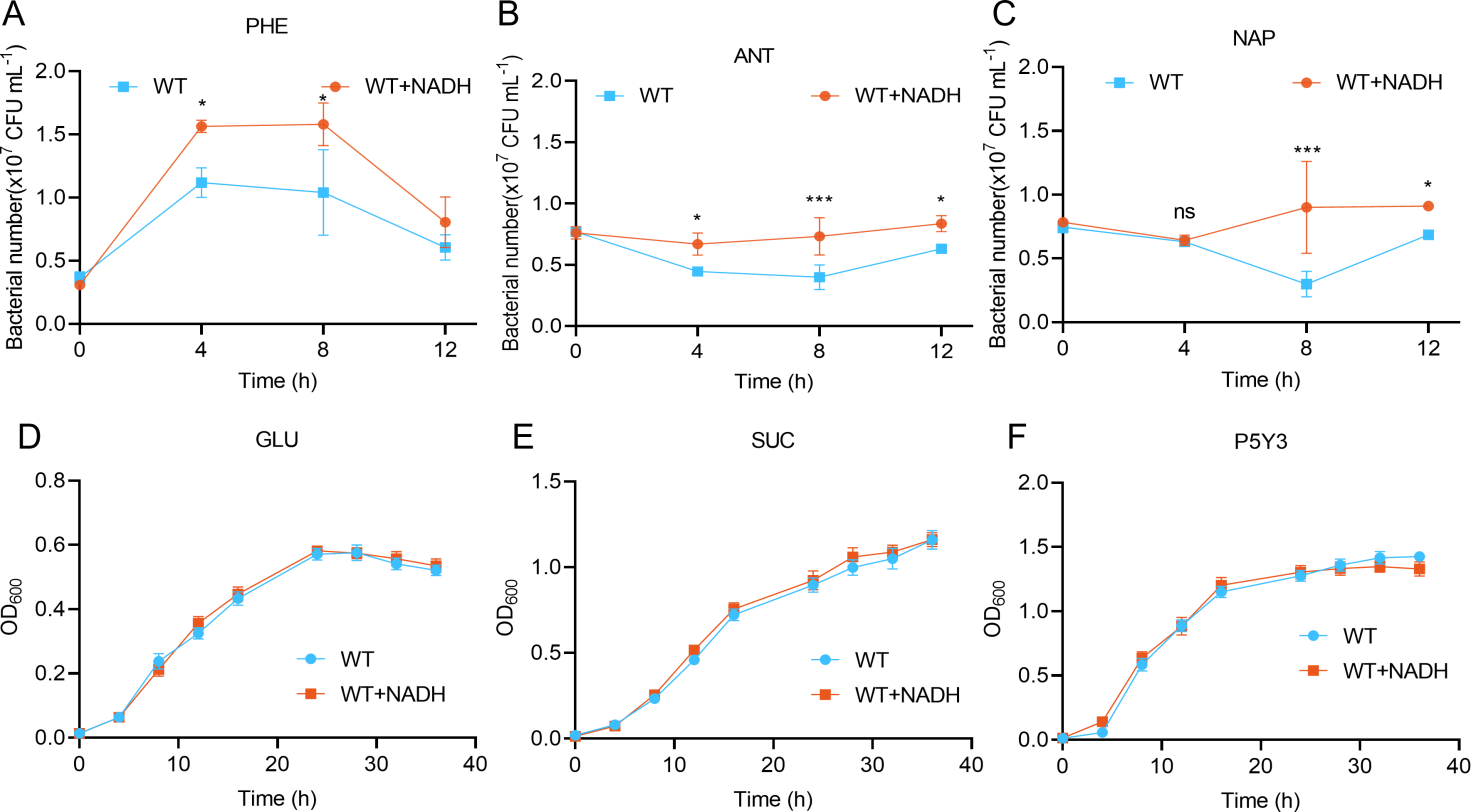
Effect of exogenous NADH on growth of US6-1 at different conditions. Growth curve of US6-1 when using (**A**)phenanthrene, (**B**)anthracene, (**C**)naphthalene, (**D**)glucose, or (**E**)sucrose as the sole carbon source or (**F**)P5Y3 media. WT: wild type, WT+NADH: wild type with exogenous NADH supplementation; PHE: phenanthrene; ANT: anthracene; NAP: naphthalene. Reported is the average of biological triplicates with the standard deviation as error bar.

When cultivated with refractory PAHs (phenanthrene, anthracene, and naphthalene) as sole carbon sources, the addition of exogenous NADH significantly increased *N. pentaromativorans* US6-1 colony counts (Fig. 1A through C) and culture turbidity after 12 h, indicating substantially enhanced bacterial growth. In contrast, NADH supplementation showed negligible effects on US6-1 growth in media containing conventional carbon sources (glucose and sucrose) or the nutrient-rich P5Y3 complex medium (Fig. 1D through F). Growth assays with NADH as the sole carbon source confirmed that strain US6-1 cannot utilize NADH for growth (Fig.S1). This demonstrates that exogenous NADH selectively promotes the utilization of PAHs as carbon substrates by US6-1.

**Fig 2:**
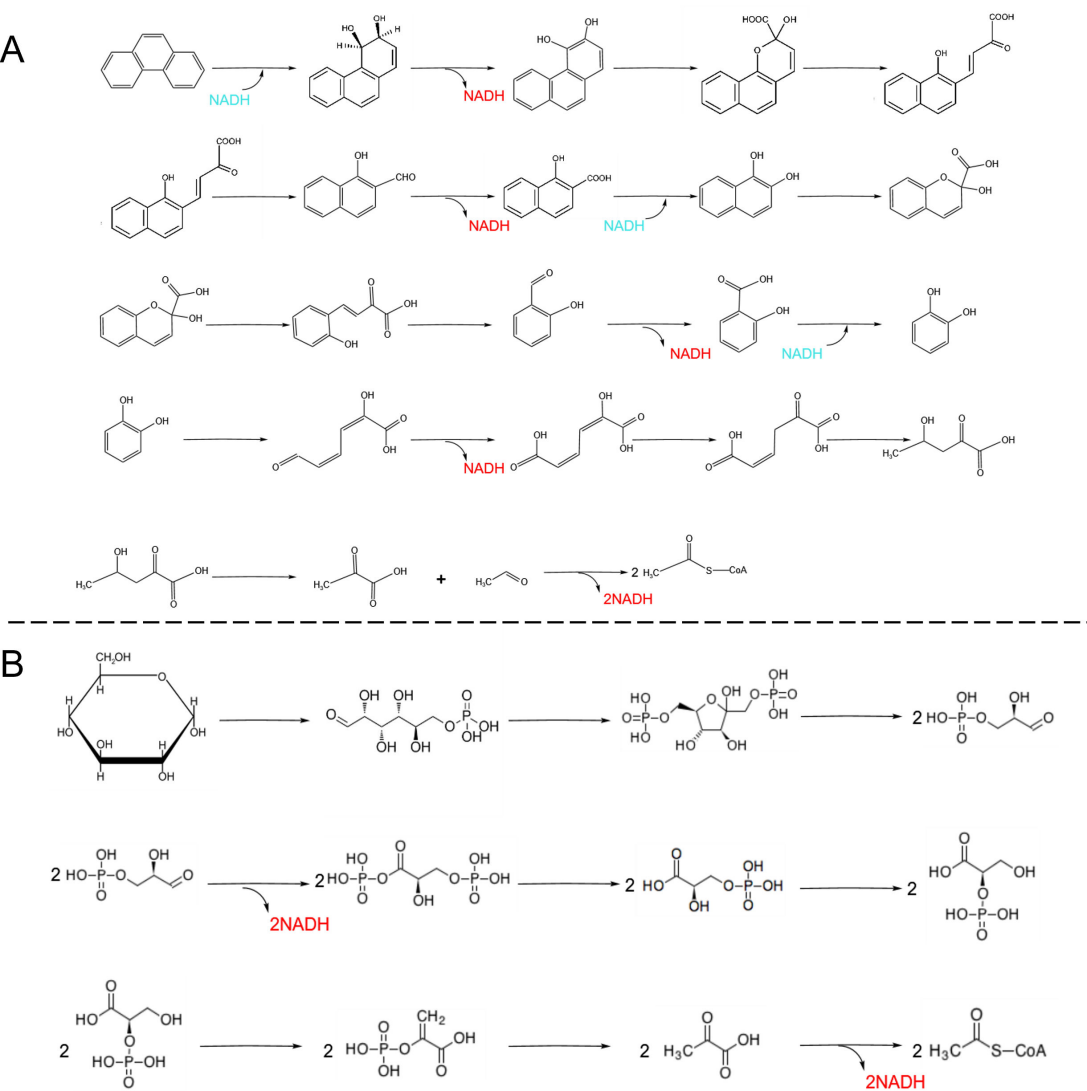
Schematic diagram of NADH consumption and production during the degradation of phenanthrene and glycolysis. (**A**)Simplified pathway of phenanthrene degradation via the 2-hydroxymuconic semialdehyde pathway, showing the consumption of 3 moles of NADH and generation of 6 moles, resulting in a net yield of 3 moles NADH per mole of phenanthrene. (**B**)Simplified glycolysis pathway for glucose, generating 4 moles of NADH with no consumption. The text highlighted in red indicates NADH production steps; the text highlighted in blue indicates NADH consumption steps.

KEGG metabolic pathway analysis suggested a potential metabolic basis for the observed substrate specificity. The predicted degradation of 1 mole of phenanthrene via the 2-hydroxymuconic semialdehyde pathway results in a net gain of 3 moles of NADH. In contrast, the catabolism of 1 mole of glucose to acetyl-CoA is predicted to yield 4 moles of NADH without net consumption (Fig. 2). While directly quantitative comparisons between pathways are complex, this simplified stoichiometric overview hints that PAH catabolism may operate with a relatively lower NADH yield per carbon unit assimilated compared to a highly efficient pathway like glycolysis. This comparison clearly demonstrates a plausible metabolic context for why NADH availability could become a more critical factor during PAH utilization.

**Fig 3:**
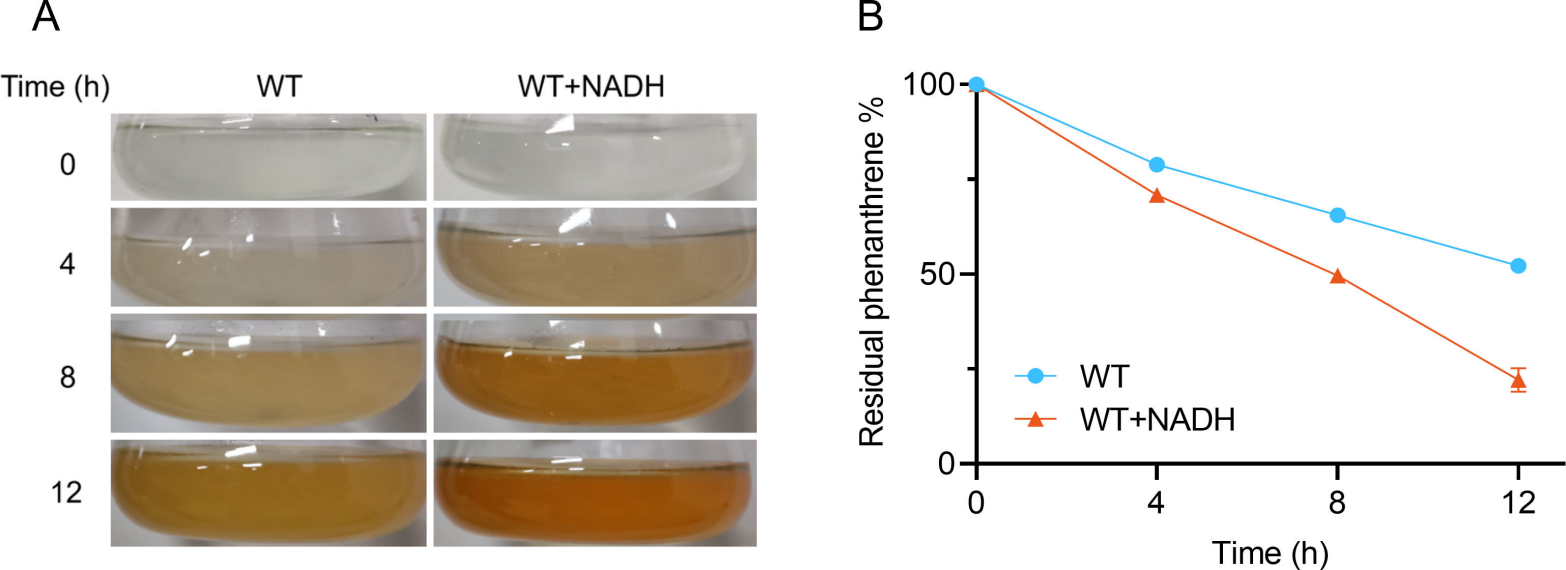
Effect of NADH on phenanthrene degradation. (**A**)Color changes during the phenanthrene degradation process by US6-1 after exogenous addition of 10 mg/L NADH. Phenanthrene degradation by US6-1 produces 2-hydroxy muconic semialdehyde, a yellow-colored compound. The higher the phenanthrene degradation rate, the greater the accumulation of this substance, resulting in a deeper yellow coloration of the culture medium. (**B**)Residual amounts during phenanthrene degradation by US6-1 with exogenous addition of 10 mg/L NADH. With an initial phenanthrene content of 100%, the residual amount of phenanthrene was measured every 4 h. WT: wild type; WT+NADH: wild type with NADH supplementation. Statistical significance between the control (WT) and NADH-supplemented group at each time point was determined using an unpaired two-tailed Student’s *t*-test. Error bars represent the standard deviation (SD) of biological triplicates. *: *P* < 0.05; ***: *P* < 0.001.

Given this perspective, we hypothesized that intracellular NADH levels might limit PAH degradation. To test this, the addition of exogenous NADH on phenanthrene degradation was investigated. We found that 10 mg/L NADH is the most effective concentration for enhancing phenanthrene degradation rates (Fig.S2), and additional NADH indeed promotes the phenanthrene degradation by faster darkening of the culture medium (Fig. 3A). Time-course analysis confirmed significantly lower residual phenanthrene levels in NADH-supplemented cultures at 4, 8, and 12 h, corresponding to degradation rate increases of 14.0%, 26.2%, and 57.2%, respectively, compared with the controls without NADH addition (Fig. 3B). In line with a functional role specific to PAH metabolism, exogenous NADH did not alter intracellular NAD(H) (NADH and NAD^+^) pools in glucose-grown cells (Fig.S3A) but increased them by 15% in phenanthrene-grown cells (Fig.S3B).

### Reduced NADH levels impair phenanthrene degradation and growth

**Fig 4:**
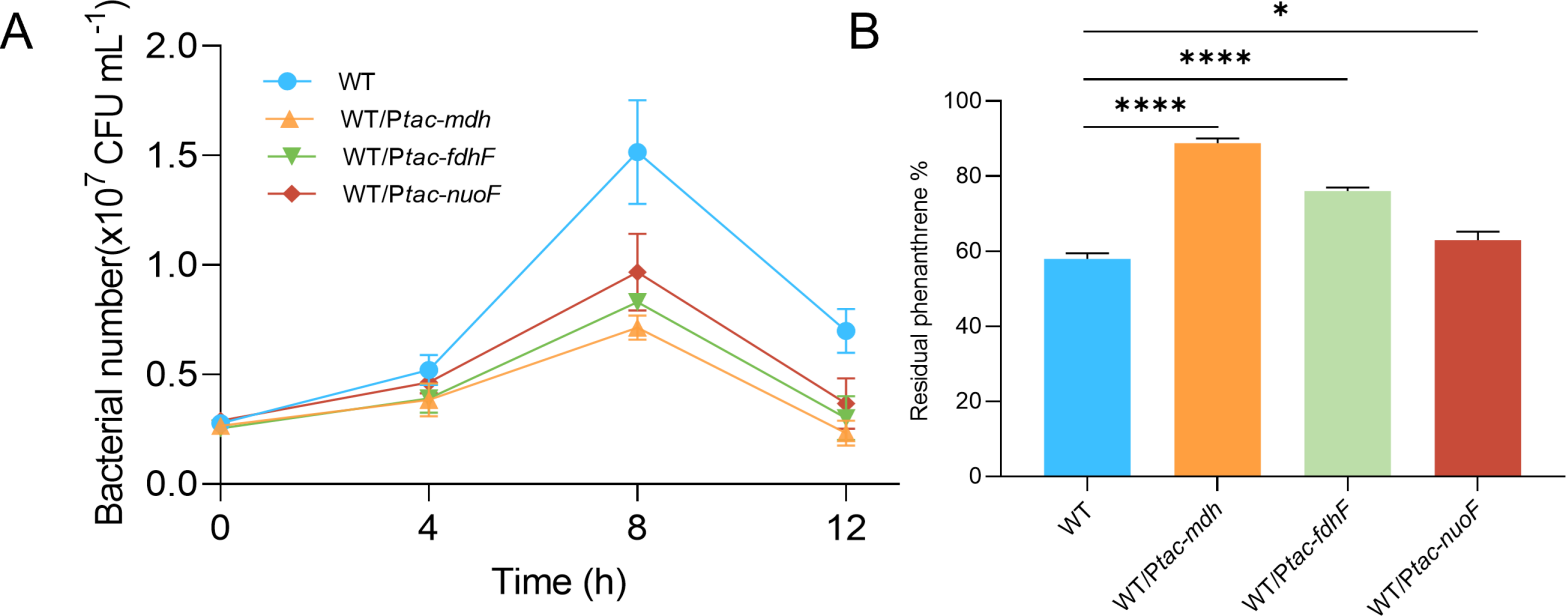
Effect of overexpression of *mdh*, *fdhF*, and *nuoF* on the growth of US6-1 and its phenanthrene degradation. (**A**)Effects of overexpressing *mdh*, *fdhF*, and *nuoF* on the growth of US6-1 when grown with phenanthrene as the sole carbon source. (**B**)Effect of overexpression of *mdh*, *fdhF*, and *nuoF* on phenanthrene degradation. WT: US6-1 wild type; WT/P*tac-mdh*: US6-1 overexpressing *mdh*; WT/P*tac-fdhF*: US6-1 overexpressing *fdhF*; WT/P*tac-nuoF*: US6-1 overexpressing *nuoF*. The expression of target genes was controlled with 0.2 mM IPTG. *: *P* < 0.05; ****: *P* < 0.0001.

To investigate whether endogenous NADH availability directly influences phenanthrene degradation, genes encoding NADH-consuming enzymes (*mdh*[24, 25] for malate dehydrogenase, *fdhF*[26, 27] for formate dehydrogenase, and *nuoF*[28] for quinone oxidoreductase subunit) were overexpressed in *N. pentaromativorans* US6-1, generating strains WT/P*tac-mdh*, WT/P*tac-fdhF*, and WT/P*tac-nuoF*. Compared with the wild-type strain (WT), these strains exhibited significantly lower intracellular NADH levels (decreased by 25%, 19%, and 30%, respectively) (Fig.S4). Consequently, while all overexpression strains displayed slower growth than the WT with phenanthrene as the sole carbon source (Fig. 4A), they showed no significant growth impairment in glucose minimal medium (Fig.S5). After 9 h of degradation, residual phenanthrene levels in WT/P*tac-mdh*, WT/P*tac-fdhF*, and WT/P*tac-nuoF* were 52%, 30%, and 10% higher, respectively, compared with the WT (Fig. 4B). These results demonstrate that depleting endogenous NADH via overexpression of NADH-consuming enzymes directly reduces phenanthrene degradation and impairs bacterial growth.

### Exogenous NADH differentially regulates key phenanthrene-degrading enzymes at the transcription level

**Fig 5:**
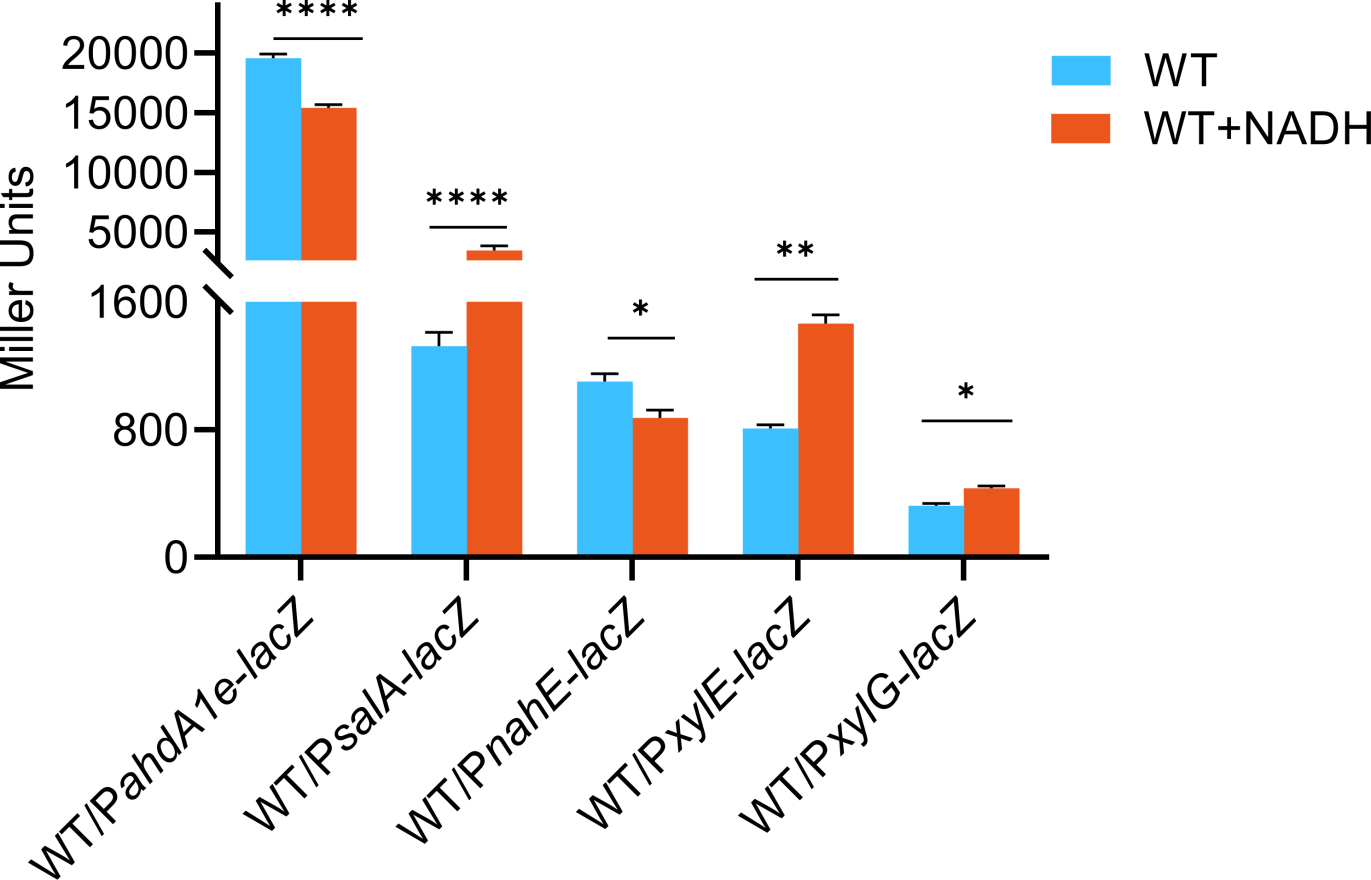
Effect of exogenous NADH addition on promoter activity of genes encoding phenanthrene-degrading enzymes. The blue group serves as the control group for measuring the promoter activity of each gene without NADH addition. The orange group was the experimental group for detecting the promoter activity of each gene after NADH supplementation. *: *P* < 0.05; **: *P* < 0.01; ****: *P* < 0.0001.

**Fig 6:**
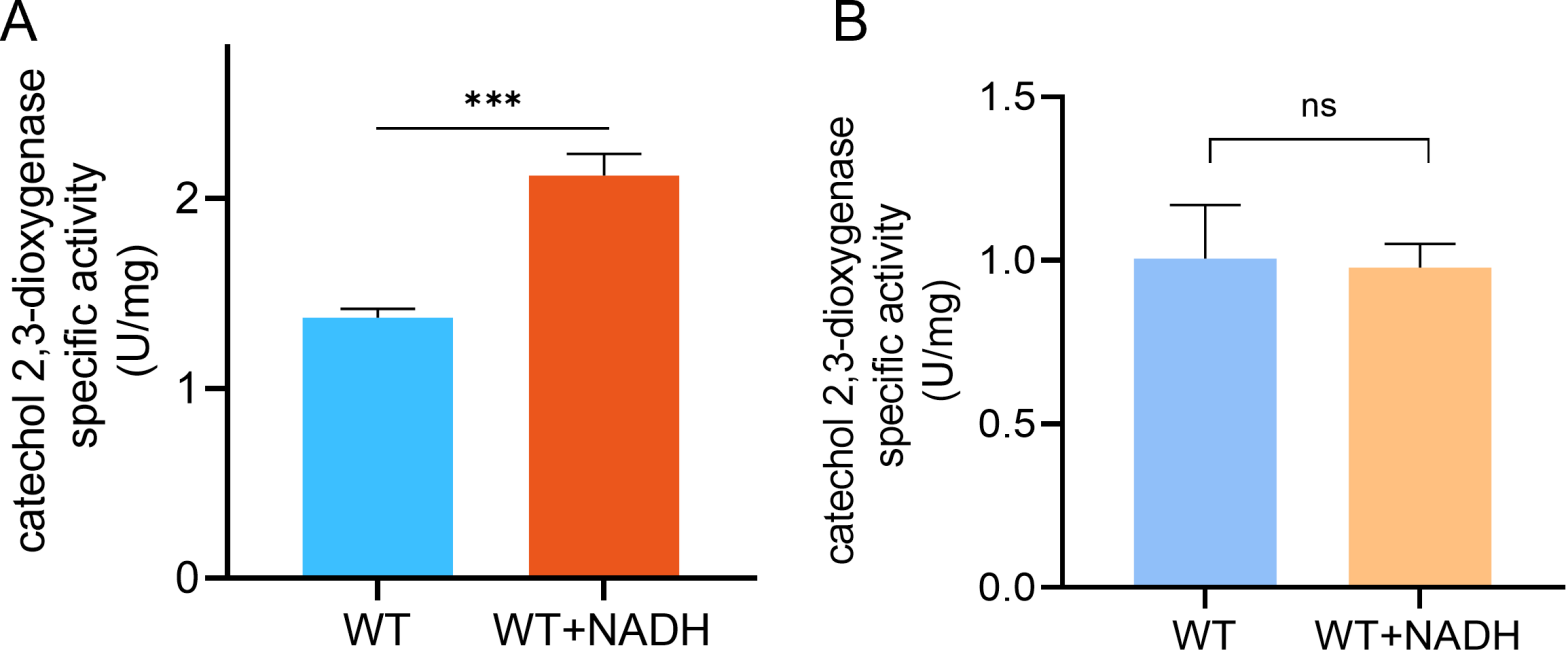
Effect of NADH on XylE activity. (**A**)Changes in XylE activity when US6-1 was cultured to the log phase, followed by exogenous addition of NADH in the culture medium. (**B**)Effect of NADH supplementation on the activity of crude XylE. WT: wild type, WT+NADH: wild type with NADH added. ns: no significant difference; ***: *P* < 0.001.

Analysis of the phenanthrene degradation pathway in US6-1 (Fig.S6) confirmed involvement of NADH in multiple redox steps. We therefore investigated whether exogenous NADH influences the expression and activity of key pathway enzymes: initial dioxygenase (AhdA1e), hydratase-aldolase (NahE), salicylate hydroxylase (SalA), catechol-2,3-dioxygenase (XylE), and 2-hydroxymuconic semialdehyde dehydrogenase (XylG). Promoter activity assays revealed that exogenous NADH significantly increased promoter activities of *salA* (158%), *xylE* (81%), and *xylG* (33%), while decreasing promoter activities of *ahdA1e* (21%) and *nahE* (32%) (Fig. 5). This gene-specific regulation demonstrates that NADH’s effect is not determined by whether an enzyme directly utilizes NADH as a cofactor, suggesting an indirect regulatory mechanism. Consistent with the transcription upregulation of *xylE*, exogenous NADH supplementation during early culture increased *in vivo* XylE enzyme activity by 35% (Fig. 6A). Remarkably, adding NADH directly to cell-free XylE crude extracts had no effect on enzyme activity (Fig. 6B). These results indicate that NADH enhances XylE activity at least in part by boosting its expression level. While a direct cofactor role was ruled out by the cell-free assay, we cannot exclude the possibility that NADH also influences XylE activity indirectly through post-translation modifications or by optimizing the intracellular redox environment needed for enzyme activity.

### Exogenous NADH relieves phenanthrene-induced oxidative stress

**Fig 7:**
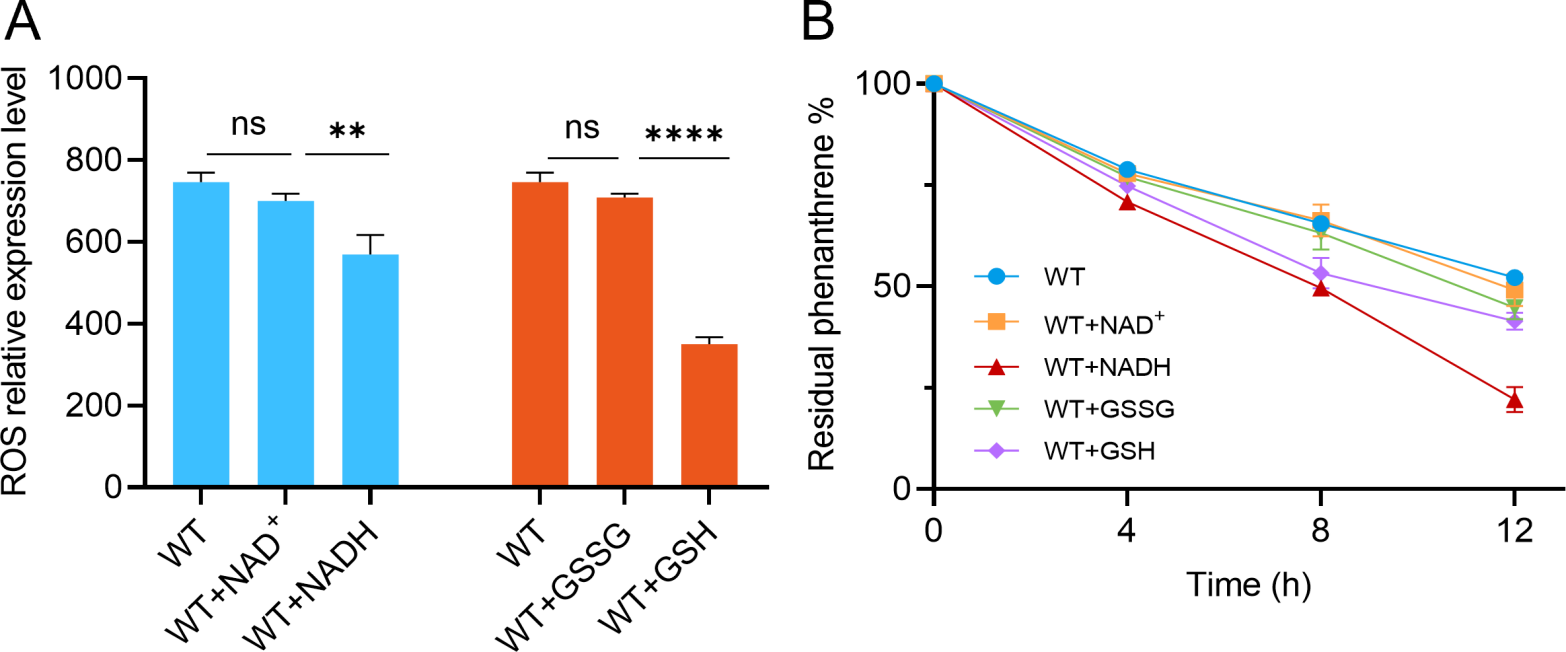
Effect of oxidizing and reducing substances on phenanthrene degradation. (**A**)Effect of exogenous GSSG, GSH, NADH, and NAD^+^ on intracellular ROS levels in US6-1 when grown with phenanthrene as the sole carbon source. (**B**)When phenanthrene was used as the sole carbon source, the residual phenanthrene in the culture medium was measured at 4, 8, and 12 hafter exogenous addition of GSSG, GSH, NADH, and NAD^+^. WT: wild type; WT+NADH: wild type with added NADH; WT+NAD^+^: wild type with added NAD^+^; WT+GSSG: wild type with added GSSG; WT+GSH: wild type with added GSH. ns: no significant difference; **: *P* < 0.01; ****: *P* < 0.0001.

**Fig 8:**
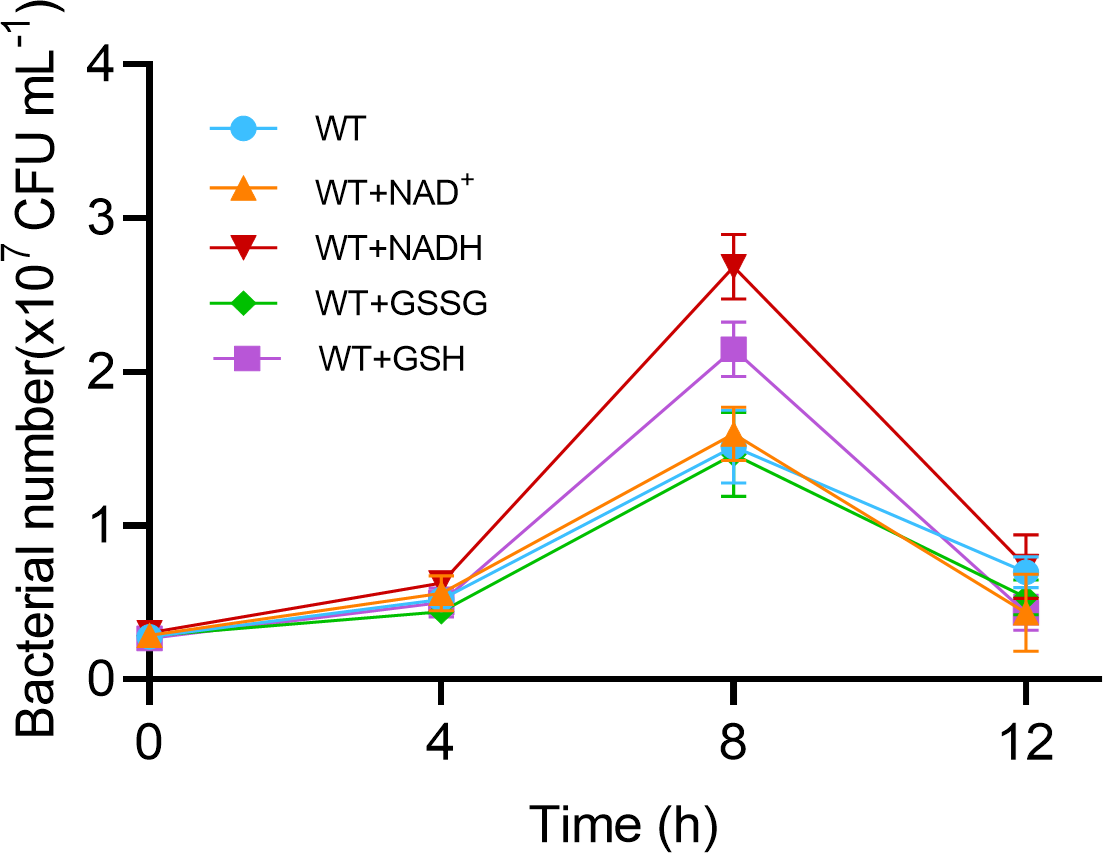
Effect of exogenous oxidizing and reducing substances on US6-1 growth when phenanthrene was used as carbon source. Bacterial growth was monitored every 4 h using the colony counting method. WT: wild type; WT+NADH: wild type with added NADH; WT+NAD^+^: wild type with added NAD^+^; WT+GSSG: wild type with added GSSG; WT+GSH: wild type with added GSH.

US6-1 growth on phenanthrene had significantly higher intracellular reactive oxygen species (ROS) levels (~100% increase across logarithmic, stationary, and death phases) compared with growth on glucose (Fig.S7), reflecting the inherent oxidative stress of PAH metabolism in strain US6-1. Importantly, exogenous NADH supplementation reduced intracellular ROS levels by 30% in phenanthrene-grown cells (Fig.S8), suggesting NADH’s role in alleviating oxidative stress due to the reduction capacity of NADH. To confirm this, comparison of NADH with other redox molecules to affect ROS level and phenanthrene degradation was performed. NADH reduced ROS by 23% and increased the phenanthrene degradation rate by 60% at 12 h, while glutathione (GSH) reduced ROS by 53% but only increased the phenanthrene degradation rate by 21%. Conversely, oxidizing substances (GSSG and NAD^+^) had negligible effects on both ROS levels and phenanthrene degradation rates (Fig. 7A and B). This indicates that while ROS scavenging contributes to NADH’s benefit, its superior enhancement of phenanthrene degradation suggests additional mechanisms beyond its antioxidant activity. In addition, exogenous NADH and GSH also significantly enhanced US6-1 growth on phenanthrene (Fig. 8), further linking ROS mitigation to bacterial fitness during PAH utilization.

### Exogenous NADH enhances phenanthrene degradation by boosting electron transport system (ETS) activity

**Fig 9:**
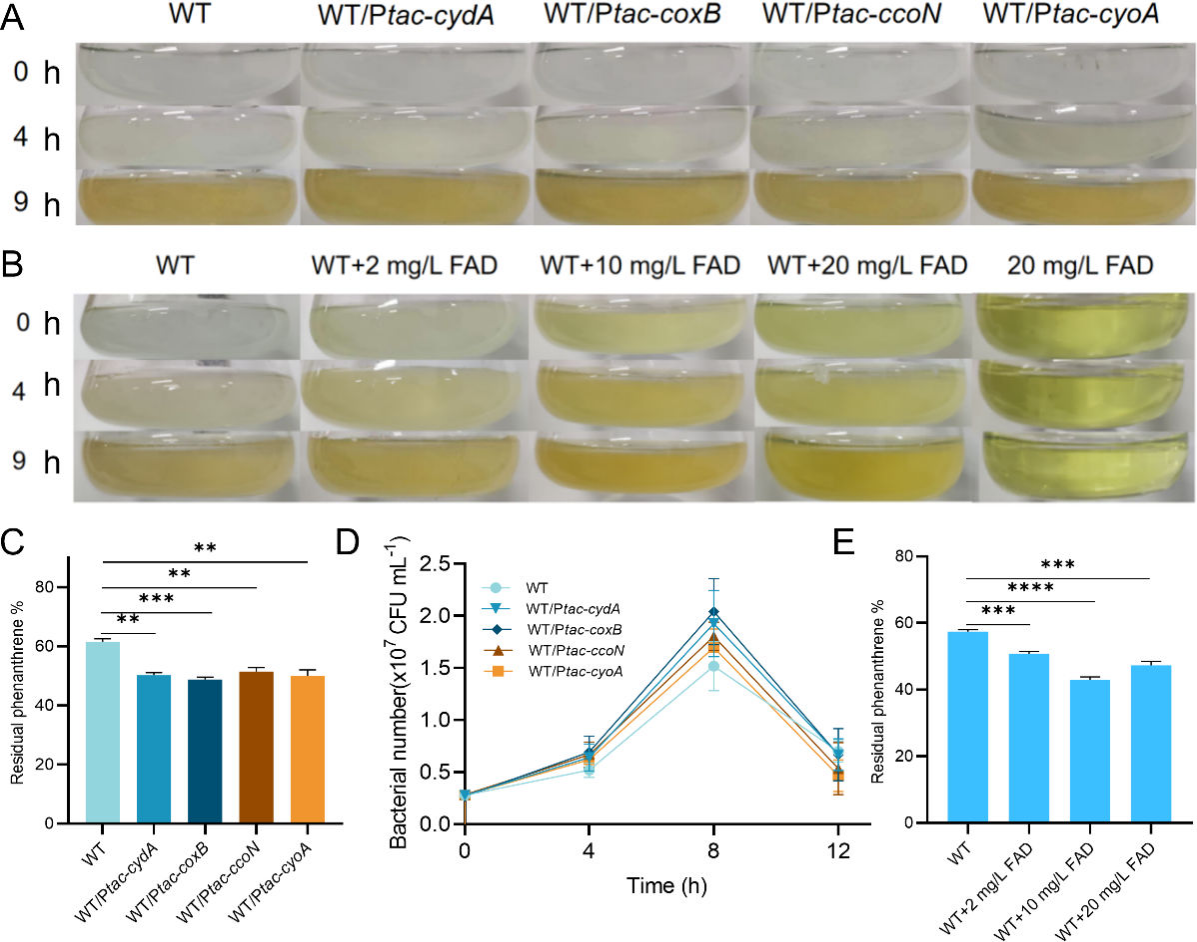
Effect of overexpression of respiratory terminal oxidase subunits and FAD supplementation on phenanthrene degradation. (**A**)Color changes during phenanthrene degradation by the five strains: WT, WT/P*tac-cydA*, WT/P*tac-coxB*, WT/P*tac-ccoN*, and WT/P*tac-cyoA*. (**B**)Color changes during phenanthrene degradation by wild-type US6-1 with different concentrations of FAD. (**C**)Residual phenanthrene after 9 h of degradation by the five strains: WT, WT/P*tac-cydA*, WT/P*tac-coxB*, WT/P*tac-ccoN*, and WT/P*tac-cyoA*. (**D**)Effect of overexpressing *cydA*, *coxB*, *ccoN*, and *cyoA* on cell growth when phenanthrene was used as the sole carbon source. (**E**)Residual phenanthrene during 200 mg/L phenanthrene degradation by wild-type US6-1 with different concentrations of FAD. **:*P*< 0.01；***：*P*< 0.001; ****: *P* < 0.0001.

The effect of NADH supplementation on ETS activity was investigated. It was found that exogenous NADH significantly enhanced EST activity of US6-1 when grown on phenanthrene as the sole carbon source, while it had negligible effect on ETS activity of US6-1 when grown on glucose as the sole carbon source (Fig.S9). This substrate-specific enhancement suggests a critical role of NADH for elevated electron flux involved in PAH degradation. To confirm this, genes (*cydA*, *coxB*, *ccoN*, and *cyoA*) encoding terminal oxidase subunits involved in ETS function were overexpressed in US6-1 to generate strains WT/P*tac-coxB*, WT/P*tac-ccoN*, WT/P*tac-cyoA*, and WT/P*tac-cydA*. Results in Fig.S10 and Fig. 9 showed that WT/P*tac-coxB*, WT/P*tac-ccoN*, and WT/P*tac-cyoA* all increased ETS activities and phenanthrene degradation. Interestingly, WT/Ptac-*cydA* also enhanced phenanthrene degradation but reduced ETS activity, indicating potential compensatory effect or distinct role for specific oxidase in PAH metabolism. To mimic NADH’s effect on electron carriers, flavin adenine dinucleotide (FAD), a key electron carrier (accepting electrons/hydrogen ions to form FADH_2_), was supplemented. It was found that exogenous FAD also significantly improved phenanthrene degradation. After 9 h, cultures with 2, 10, and 20 mg/L FAD showed darker coloration (indicating faster degradation) and reduced residual phenanthrene levels by 11%, 24%, and 17%, respectively, compared with the controls of no FAD addition (Fig. 9). This demonstrates that facilitating electron flux through the ETS by NADH directly enhances phenanthrene catabolism.

## DISCUSSION

This study elucidated the multifaceted role of exogenous NADH in enhancing phenanthrene degradation in *N. pentaromativorans* US6-1. Exogenous NADH specifically promoted US6-1 growth and phenanthrene degradation only when PAHs served as the sole carbon source but exhibited negligible effect when conventional carbon sources like glucose or nutrient-rich media were consumed. This substrate specificity, together with KEGG pathway analysis, revealed that compared with glucose catabolism, phenanthrene catabolism yields less NADH per assimilated carbon unit, suggesting PAH metabolism creates a higher cellular demand for this cofactor.

Exogenous NADH significantly accelerated phenanthrene degradation kinetics and increased intracellular NADH levels. Genetic depletion of endogenous NADH via overexpression of NADH-consuming enzyme genes (*mdh*, *fdhF*, and *nuoF*) impaired both growth and degradation efficiency, directly establishing the essential role of NADH availability in PAH catabolism. This finding highlights the potential of modulating NADH levels to influence PAH bioremediation.

Our investigation revealed three interconnected mechanisms underpinning NADH’s effect on phenanthrene degradation. First, NADH differentially regulated key phenanthrene-degrading enzymes, significantly upregulating *salA*, *xylE*, and *xylG* promoter activity while downregulating *ahdA1e* and *nahE*. This gene-specific effect, which is uncorrelated with direct enzyme cofactor requirements (29, 30), suggests indirect metabolic flux control. Consistent with transcription upregulation, NADH enhanced *in vivo* XylE activity (+35%) but had no effect on cell-free extracts, endorsing the regulatory effects of NADH on the transcription level of *xylE*. It is important to acknowledge that other factors like post-translation modulation and metabolic environments could also be involved in this process.

Besides, phenanthrene degradation induced significantly higher intracellular ROS levels than glucose metabolism (~100% increase). Exogenous NADH effectively reduced this oxidative burden (−30%). While the potent reductant GSH reduced ROS more effectively (−53%), it enhanced degradation less significantly than NADH (+21%vs +60% at 12 h). This disparity implies that while ROS scavenging contributes to NADH’s benefit, its superior degradation enhancement relies on complementary mechanisms. Both reductants (NADH and GSH) also improved growth on phenanthrene, linking reduced oxidative stress to improved cellular fitness.

Finally, NADH specifically boosted ETS activity by 50% during phenanthrene degradation but had no effect during glucose utilization. Interestingly, overexpression of most terminal oxidase genes (*coxB*, *ccoN*, and *cyoA*) increased both ETS activity and degradation rate, supporting the critical role of electron flux. Similarly, the electron carrier FAD, which facilitates ETS flux, also accelerated phenanthrene degradation. All these results demonstrate that NADH promotes phenanthrene degradation, in part, by stimulating electron flux through the ETS.

The dual mechanisms of ETS enhancement and oxidative stress alleviation are likely interlinked rather than independent. We proposed that the increased NADH pool primarily serves to boost the capacity and efficiency of the electron transport chain (ETC). The observed boost in ETS activity (Fig.S9) implies a more efficient electron flow, which is known to minimize electron leakage. Therefore, exogenous NADH likely boosts the ETC activity, promoting efficient electron flux and reducing electron leakage that typically lead to ROS generation and providing the reducing power necessary for antioxidant systems (like glutathione reductase, indirectly) to scavenge ROS. This explains why NADH promotes degradation more effectively than GSH (which only scavenges ROS).

In conclusion, this study provides compelling evidence that NADH significantly enhances PAH degradation in *N. pentaromativorans* US6-1 through a synergistic interplay of mechanisms: (i) regulating the expression and activity of key catabolic enzymes, (ii) alleviating phenanthrene-induced oxidative stress, and (iii) boosting electron flux in the respiratory chain. The substrate-specific nature of this enhancement is explained by the higher NADH demand inherent to PAH catabolism compared to conventional carbon sources. While offering novel insights into NADH’s regulatory roles in PAH biodegradation and demonstrating the feasibility of manipulating NADH levels as a bioremediation strategy, this work represents an initial exploration. Further research is warranted to fully dissect the molecular pathways of NADH’s gene regulation and its precise interplay with redox homeostasis and energy metabolism.

## MATERIALS AND METHODS

### Bacterial strains, plasmids, and culture conditions

Table S1 lists all bacterial strains and plasmids that were utilized in this study. *Escherichia coli* WM3064 was cultured in LB medium at 37°C supplemented with 0.3 mM diaminopimelic acid (DAP). *N. pentaromativorans* US6-1 was cultured in P5Y3 medium (containing 25 g/L sea salt, 5 g/L tryptone LP0137, 3 g/L yeast extract) at 30°C. During biparental conjugation between *N. pentaromativorans* US6-1 and *E. coli* WM3064, the conjugated strains were cultured in a medium containing equal parts P5Y3 and LB at 30°C, and supplemented with 0.3 mM DAP.

### Construction of gene overexpression mutants

In this study, all overexpression strains were constructed using the pHGE-P*tac* plasmid, and the recombinant strains were generated through the one-step cloning method. The target fragment was amplified by PCR and cloned into the pHGE-P*tac* plasmid. This recombinant plasmid was transformed into *E. coli* WM3064 and then introduced into *N. pentaromativorans* US6-1 via conjugation. Sequencing verification confirmed the successful construction of the overexpression strain.

### Construction of the reporting system strains

First, approximately 300–500 bp promoter region fragments were amplified via PCR and then purified. Next, the pHGEI03 plasmid was linearized and purified. Subsequently, one-step cloning was used to ligate the target fragment with the linearized plasmid, and the ligation product was transformed into *E. coli* WM3064, followed by screening via colony PCR and sequencing verification. Next, the recombinant plasmid was introduced into US6-1 using biparental conjugation. After initial screening on P5Y3 plates containing kanamycin resistance, the target strain was further identified through PCR verification and sequencing.

### Bacterial growth detection by colony counting method

First, single colonies were picked and cultured overnight in P5Y3 medium. Then, the bacterial suspension was inoculated at a 1:100 ratio into a medium with phenanthrene as the sole carbon source for cultivation. Samples were taken every 4 h, and the bacterial suspension was diluted 10-fold before spreading 100 μL of the suspension onto plates, with each sample repeated in triplicate. After incubating at 30°C for 48 h, the colony counts on the medium were recorded.

### Determination of phenanthrene content

After overnight culture of the degrading bacteria in P5Y3 medium, cell culture was transferred to a medium with phenanthrene as the sole carbon source and incubated at 30°C with shaking for a specified duration. An equal volume of the bacterial culture with ethyl acetate was vortexed to ensure thorough contact. The mixture was transferred to a separatory funnel and allowed to stand for phase separation. Next, the lower aqueous phase was collected into a round-bottom flask. An equal volume of ethyl acetate was added and the extraction was repeated once. The aqueous phase was treated with anhydrous sodium sulfate to remove impurities, and the organic phases from the three extractions were combined into the same round-bottom flask. The contents of the flask were concentrated by rotary evaporation until the ethyl acetate was completely evaporated. The residual phenanthrene in the flask was resolved using methanol and measured using high-performance liquid chromatography (HPLC) (31).

### Detection of catechol-2,3-dioxygenase activity

When the test bacterial strain reaches the logarithmic growth phase, the bacterial suspension was centrifuged at 8,000 rpm for 2 minat 4°C. After centrifugation, the supernatant was removed, and the bacterial pellet was resuspended in PBS buffer. After repeating this step twice, the liquid was ultimately collected. Subsequently, the cells were lysed using an ultrasonic disruptor. Upon completion of lysis, the solution was centrifuged again at 8,000 rpm for 2 min, and the supernatant was retained for later use. Next, the supernatant was mixed with 0.1 M catechol in a 1:1 ratio to allow the reaction generating the yellow compound 2-hydroxymuconic semialdehyde. Since this compound exhibits maximum absorbance at 375 nm, the optical density (OD_375_) was measured.

### Detection of system activity reporting

Under a constant temperature of 4°C, the logarithmic-phase bacterial sample was centrifuged at 8,000 rpm for 2 minto collect the bacterial cells. Subsequently, the bacterial cells were washed twice with PBS buffer, and the cells were resuspended. Then, an ultrasonic disruptor was used to lyse the cells. After complete lysis, the solution was centrifuged again at 8,000 rpm for 2 min, and the supernatant was collected for later use. Next, the lysate was mixed with 2-nitrophenyl *β*-D-galactopyranoside (ONPG) in 1:1 ratio, and the mixture was placed in a 96-well plate. Once the reaction solution turns yellow, its absorbance at a wavelength of 420 nm (OD_420_) was immediately measured(32).

### Determination of NADH content

Approximately 1×10^6^ cells were collected and centrifuged at 600 rpm for 5 min. The culture medium was completely aspirated, and 200 μL of ice-cold NAD^+^/NADH extraction buffer (NAD^+^/NADH Assay Kit, Beyotime, China) was added using a pipette to gently lyse the cells. Subsequently, the mixture was centrifuged at 4°C and 12,000 rpm for 5–10 min, and the supernatant was collected as the test sample. A volume of 50–100 μL of the sample was transferred to a centrifuge tube and incubated in a 60°C water bath for 30 min. Then, 20 μL of the test sample was pipetted into a 96-well plate, followed by the addition of alcohol dehydrogenase working solution, and incubated at 37°C in the dark. Afterward, 10 μL of chromogenic solution was added to the 96-well plate, mixed thoroughly, and further incubated at 37°C in the dark. Once the sample turned orange-yellow, the absorbance at 450 nm was measured.

### Detection of ROS content

2,7-Dichlorodihydrofluorescein diacetate (DCFH-DA) Dilute DCFH-DA (Beyotime ROS Detection Kit, Beyotime, China) was diluted with PBS solution at a ratio of 1:1,000 to a final concentration of 10 μM. The cells were collected and suspended in the diluted DCFH-DA. The samples were incubated in a 37°C-cell culture incubator for 20 min, gently inverted, and mixed every 3–5 minto ensure sufficient contact between the probe and cells. Subsequently, the cells were washed three times with PBS solution to remove any extracellular DCFH-DA. Finally, fluorescence intensity was measured using a fluorescence microplate reader with an excitation wavelength of 488 nm and an emission wavelength of 525 nm (33).

### Detection of electron transport chain system (ETS) activity

The total electron transport system activity was measured by iodonitrotetrazolium chloride (INT) reduction assay. Then, 500 µL of the bacterial suspension was taken out and, 1 mL of 0.2% INT solution was added. The reaction was proceeded for 30 minunder light-protected condition. To terminate the reaction, 0.1 mL of methanol was added. Subsequently, the solution was centrifuged at 10,000 rpm for 5 min. The supernatant was removed, and then 1 mL of 95% methanol was added. Then, the precipitate was suspended and dissolved in the methanol. After centrifugation at 10,000 rpm for 5 min, the absorbance of the supernatant was measured at a wavelength of 495 nm (34).

## Data Availability

The data associated with this work are available from the corresponding author upon reasonable request.

## SUPPLEMENTAL MATERIAL

Supplemental material aem.00411-26-s0001.pdf Fig. S1 to S10; Table S1.

ASM does not own the copyrights to Supplemental Material that may be linked to, or accessed through, an article. The authors have granted ASM a non-exclusive, world-wide license to publish the Supplemental Material files. Please contact the corresponding author directly for reuse.
