# Supplementary material for: Exogenous NADH enhances polycyclic aromatic hydrocarbon degradation in Novosphingobium pentaromativorans US6-1 by boosting energy metabolism and reducing oxidative stress
Source: Appl Environ Microbiol. 2026 May 20;92(6):e00411-26. doi: 10.1128/aem.00411-26 (PMC13274426; doi:10.1128/aem.00411-26)
Supplement: Supplemental material — Fig. S1 to S10; Table S1. [file aem.00411-26-s0001.pdf]

Exogenous NADH Enhances Polycyclic Aromatic Hydrocarbon Degradation in *Novosphingobium pentaromativorans* US6-1 by Boosting Energy Metabolism and Reducing Oxidative Stress

Qiu Meng[a], Fengjiao Lv[a], Jiefeng Huang[a], Han Chen[a], Feifei Cao[b]*, Zhiliang Yu[a]*

[a]College of Biotechnology and Bioengineering, Zhejiang University of Technology, Hangzhou, 310014, China

[b]Hangzhou Chuhuan Science and Technology Co., Ltd., Hangzhou 310015, Zhejiang Province, China

***Corresponding authors**:

zlyu@zjut.edu.cn (Zhiliang Yu); bindk2002@126.com (Feifei Cao)

Running title: NADH Enhances PAH Degradation in *Novosphingobium*

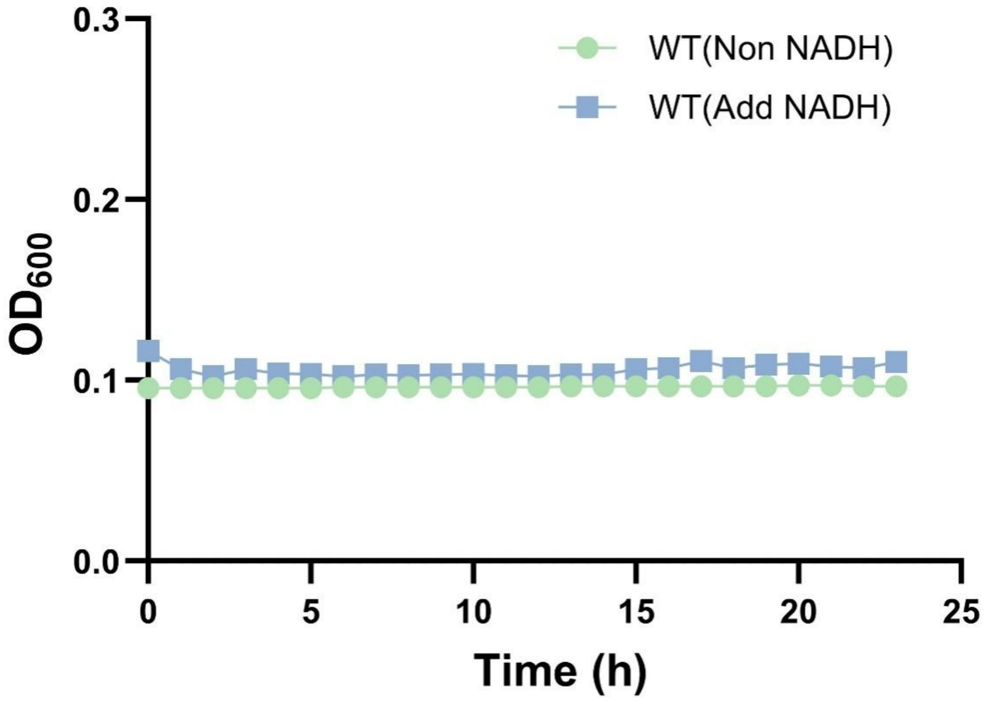

Fig. S1 Growth of strain US6-1 in minimal medium with NADH as the sole carbon source or without NADH.

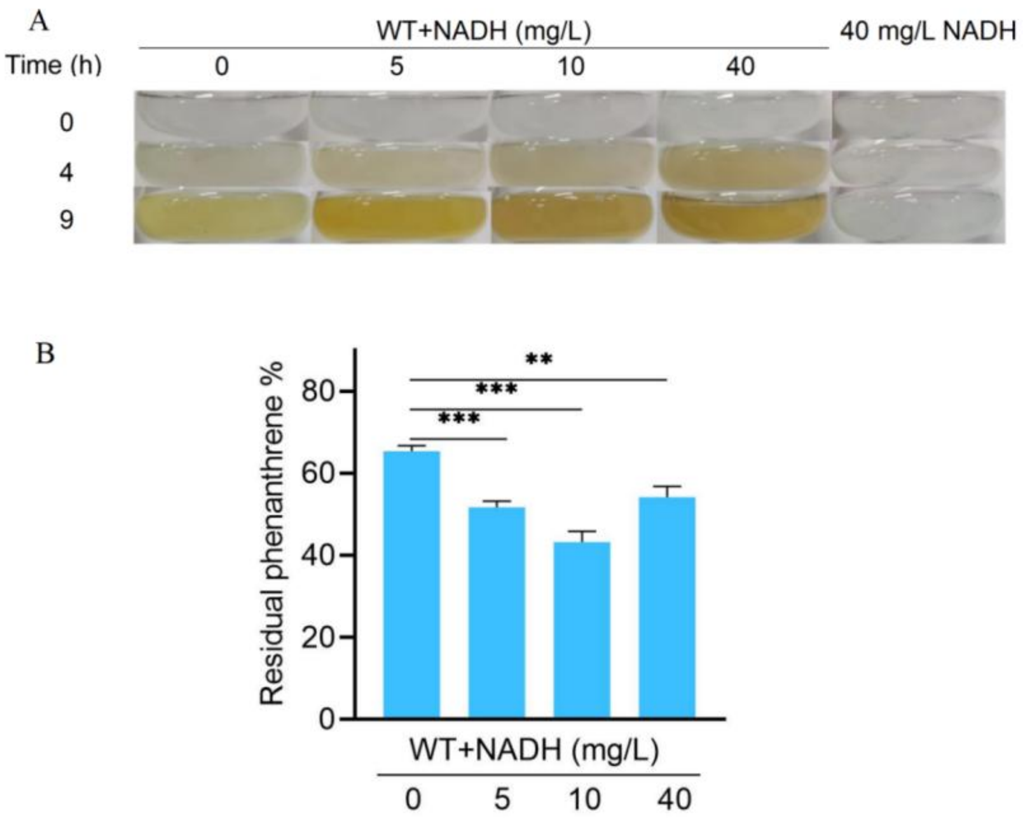

Fig. S2 Effect of exogenous NADH addition after 9 hours on the degradation of phenanthrene by US6-1. (A) Color changes during the phenanthrene degradation process by US6-1 after exogenous addition of different concentrations of NADH. During the degradation of phenanthrene by US6-1, 2-hydroxy muconic semialdehyde is produced as a yellow-colored compound. The higher the phenanthrene degradation rate, the greater the accumulation of this substance, resulting in a deeper yellow coloration of the culture medium. (B) Residual amounts during phenanthrene degradation by US6-1 with exogenous addition of different NADH concentrations. The initial phenanthrene concentration was set as 100%. WT: Wild type; WT+NADH: Wild type with NADH addition. **: P<0.01; ***: P<0.001.

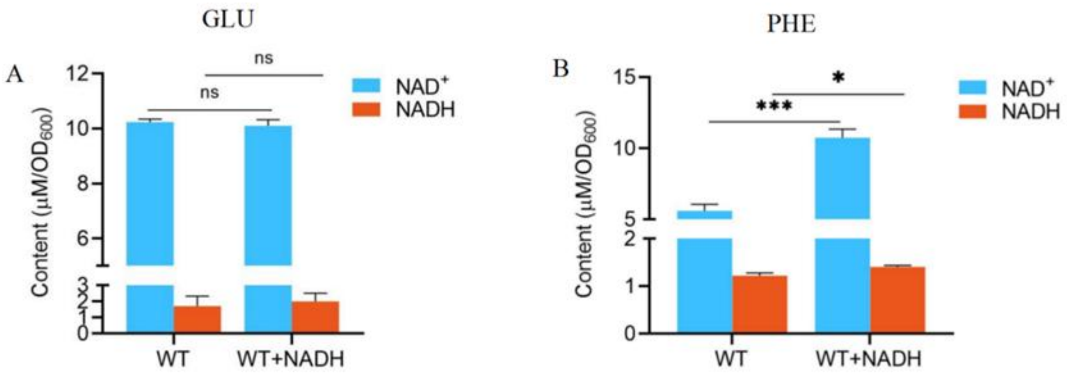

Fig. S3 Effect of exogenous NADH addition on intracellular NADH and NAD^+ content in US6-1 when glucose (A) or phenanthrene (B) was used as the carbon source. Blue bars represent NAD^+ content; red bars represent NADH content; WT: wild type; WT+NADH: wild type with NADH addition; PHE: phenanthrene; GLU: glucose. ns: not significant; *: $P < 0.05$; ***: $P < 0.001$.

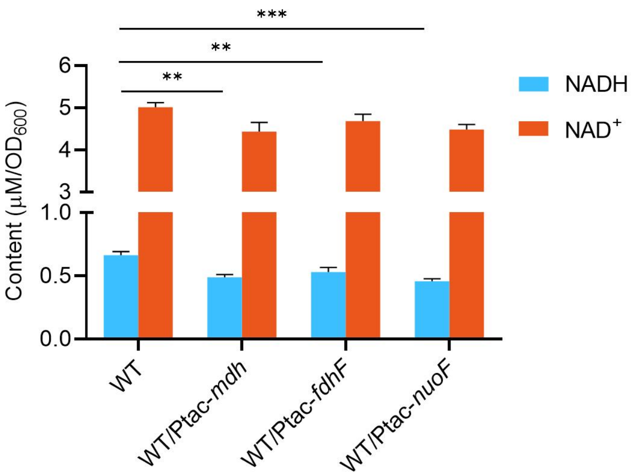

Fig. S4 Effect of overexpression of *mdh*, *fdhF*, and *nuoF* on intracellular NADH content. WT: US6-1 wild type; WT/P*tac-mdh*: Overexpression of *mdh* in US6-1; WT/P*tac-fdhF*: Overexpression of *fdhF* in US6-1; WT/P*tac-nuoF*: Overexpression of *nuoF* in US6-1. The expression of target genes was controlled by an IPTG-inducible promoter, with an IPTG concentration of 0.2 mM. **: P < 0.01; ***: P < 0.001.

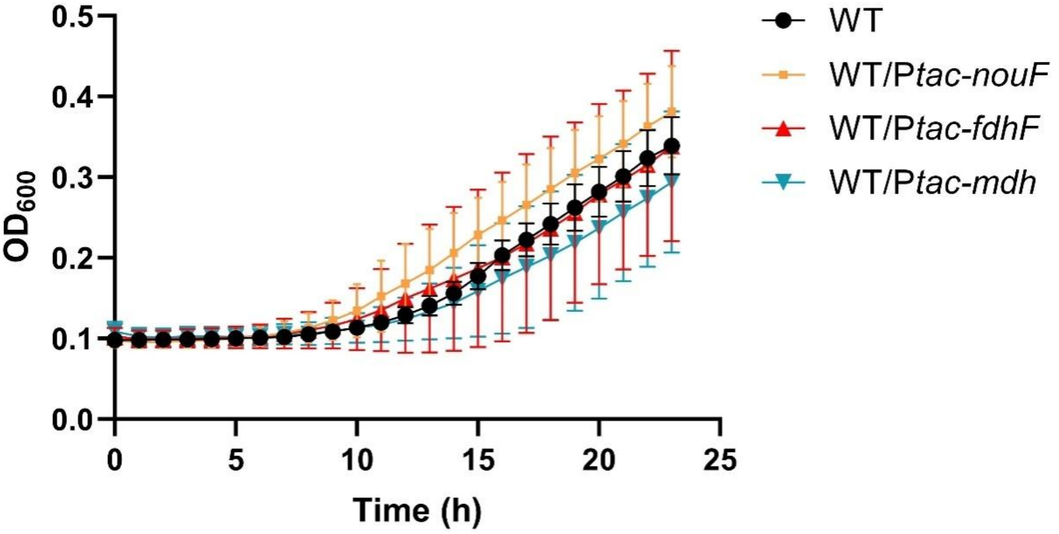

Fig. S5 Effect of overexpressing *mdh*, *fdhF*, and *nuoF* on the growth of US6-1 when grown with phenanthrene as the sole carbon source.

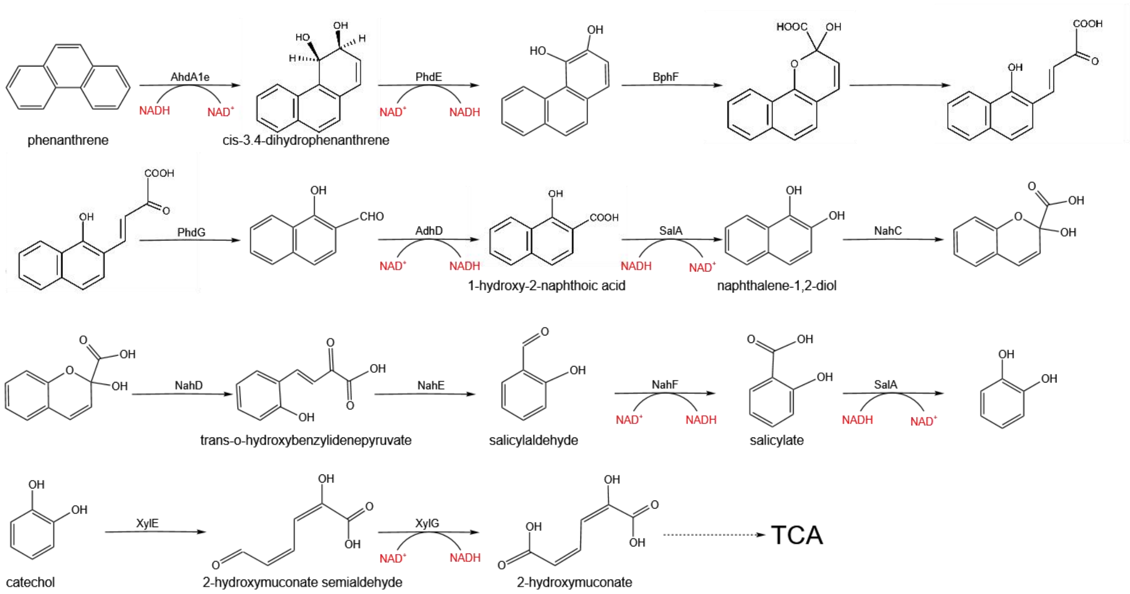

Fig. S6 Phenanthrene degradation pathway and related enzymes.

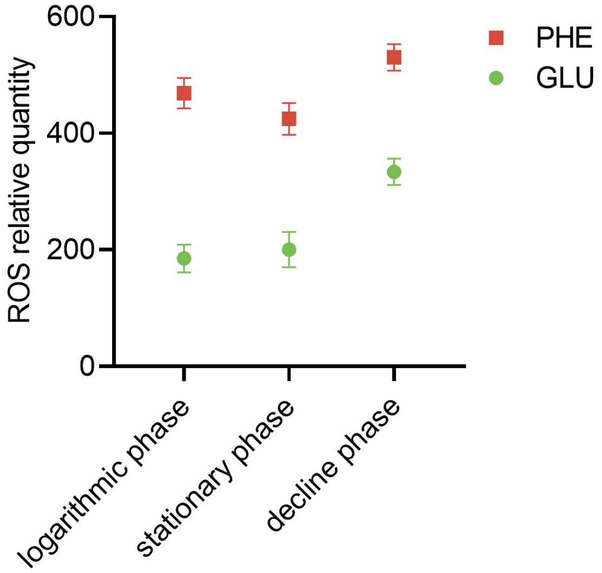

Fig. S7 Difference in ROS levels between two carbon sources (glucose and phenanthrene) used as sole carbon sources. The intracellular ROS levels of US6-1 cells at different growth phases were detected. GLU: glucose as the sole carbon source; PHE: phenanthrene as the sole carbon source.

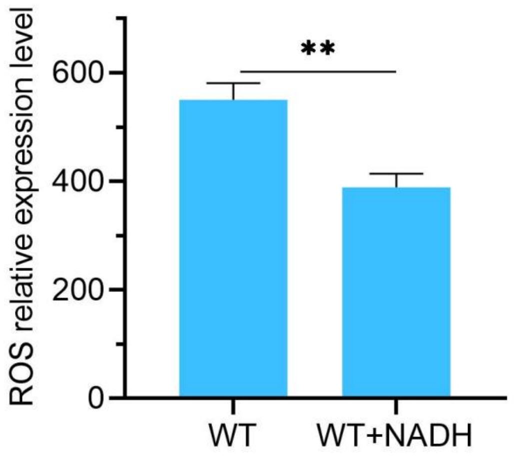

Fig. S8 Effect of NADH on ROS level during degradation of phenanthrene. This experiment was conducted in a medium with phenanthrene as the sole carbon source. WT: wild type; WT+NADH: wild type with NADH addition. **: $P < 0.01$.

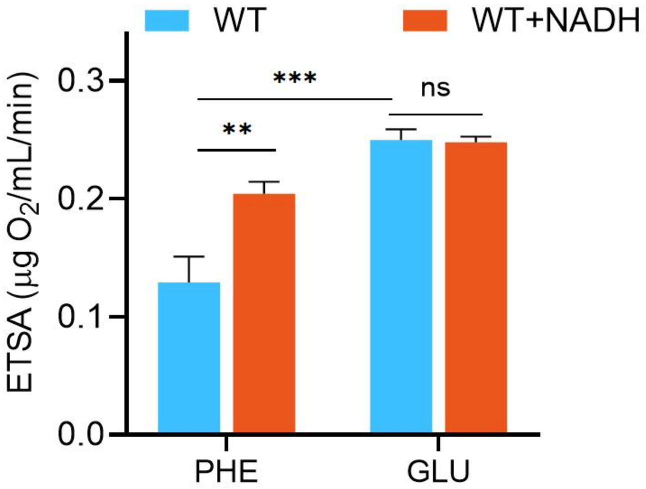

Fig. S9 Effect of adding NADH to different carbon source media on intracellular ETS activity in US6-1. WT: Wild type; WT+NADH: Wild type with NADH supplementation; ETSA: Electron transport system activity. ns: Not significant; **: P<0.01; ***: P<0.001.

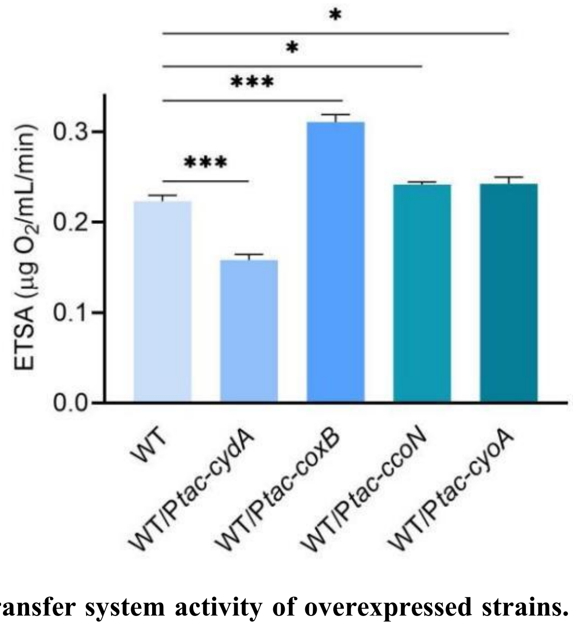

Fig. S10 Electronic transfer system activity of overexpressed strains. ETSA: electron transfer system activity. *：P< 0.05；***：P< 0.001.

Table S1 Strains and plasmids used in this study

strains or plasmids	characteristics	sources
strains		
N. pentaromativorans US6-1	wild-type (WT)	TransGenBiotech
Escherichia coli WM3064	donor bacterium	TransGenBiotech
WT/P*tac-cydA*	expressing *cydA* using the P*tac* promoter in WT	This study
WT/P*tac-coxB*	expressing *coxB* using the P*tac* promoter in WT	This study
WT/P*tac-ccoN*	expressing *ccoN* using the P*tac* promoter in WT	This study
WT/P*tac-cyoA*	expressing *cyoA* using the P*tac* promoter in WT	This study
WT/P*tac-mdh*	expressing *mdh* using the P*tac* promoter in WT	This study
WT/P*tac-fdhF*	expressing *fdhF* using the P*tac* promoter in WT	This study
WT/P*tac-nuoF*	expressing *nuoF* using the P*tac* promoter in WT	This study
WT/P*ahdA1e-lacZ*	expressing the activity of the *ahdA1e* promoter using the *lacZ* reporter system in WT	This study
WT/P*xylG-lacZ*	expressing the activity of the *xylG* promoter using the *lacZ* reporter system in WT	This study
WT/P*xylE-lacZ*	express the activity of the *xylE* promoter using the *lacZ* reporter system in WT	This study
plasmids		
pHGE-P*tac*	Km^r , IPTG-inducible P*tac* expression vector	
pHGEI03	Km^r , *lacZ* reporter vector	
